# Main causes of death in Dande, Angola: results from Verbal Autopsies of deaths occurring during 2009–2012

**DOI:** 10.1186/s12889-016-3365-6

**Published:** 2016-08-04

**Authors:** Edite Vila Nova Rosário, Diogo Costa, Luís Timóteo, Ana Ambrósio Rodrigues, Jorge Varanda, Susana Vaz Nery, Miguel Brito

**Affiliations:** 1 Health Research Centre of Angola (CISA), Caxito, Bengo Angola; 2 EPIUnit—Institute of Public Health, University of Porto (ISPUP), Porto, Portugal; 3 Hospital Geral do Bengo, Caxito, Bengo Angola; 4 National Institute of Health Doutor Ricardo Jorge, Lisboa, Portugal; 5 CRIA, Department of Life Sciences, University of Coimbra, Coimbra, Portugal; 6 Global Health and Tropical Medicine, GHTM, Instituto de Higiene e Medicina Tropical, IHMT, Universidade Nova de Lisboa, Lisboa, Portugal; 7 ANU College of Medicine, Biology and Environment, The Australian National University, Camberra, Australia; 8 Lisbon School of Health Technology (ESTeSL), Lisboa, Portugal

**Keywords:** Verbal Autopsy, Angola, Health and demographic surveillance system, Causes of death

## Abstract

**Background:**

The Dande Health and Demographic Surveillance System (HDSS) located in Bengo Province, Angola, covers nearly 65,500 residents living in approximately 19,800 households. This study aims to describe the main causes of deaths (CoD) occurred within the HDSS, from 2009 to 2012, and to explore associations between demographic or socioeconomic factors and broad mortality groups (Group I—Communicable diseases, maternal, perinatal and nutritional conditions; Group II—Non-communicable diseases; Group III—Injuries; IND—Indeterminate).

**Methods:**

Verbal Autopsies (VA) were performed after death identification during routine HDSS visits.

Associations between broad groups of CoD and sex, age, education, socioeconomic position, place of residence and place of death, were explored using chi-square tests and fitting logistic regression models.

**Results:**

From a total of 1488 deaths registered, 1009 verbal autopsies were performed and 798 of these were assigned a CoD based on the 10^th^ revision of the International Classification of Diseases (ICD-10).

Mortality was led by CD (61.0 %), followed by IND (18.3 %), NCD (11.6 %) and INJ (9.1 %). Intestinal infectious diseases, malnutrition and acute respiratory infections were the main contributors to under-five mortality (44.2 %). Malaria was the most common CoD among children under 15 years old (38.6 %). Tuberculosis, traffic accidents and malaria led the CoD among adults aged 15–49 (13.5 %, 10.5 % and 8.0 % respectively). Among adults aged 50 or more, diseases of the circulatory system (23.2 %) were the major CoD, followed by tuberculosis (8.2 %) and malaria (7.7 %). CD were more frequent CoD among less educated people (adjusted odds ratio, 95 % confidence interval for none vs. 5 or more years of school: 1.68, 1.04–2.72).

**Conclusion:**

Infectious diseases were the leading CoD in this region. Verbal autopsies proved useful to identify the main CoD, being an important tool in settings where vital statistics are scarce and death registration systems have limitations.

**Electronic supplementary material:**

The online version of this article (doi:10.1186/s12889-016-3365-6) contains supplementary material, which is available to authorized users.

## Background

Mortality data are essential for defining and evaluating public health policies and for inferring about the health status of the population in a country [1, 2]. However, medical certification of cause of death is used in only one third of deaths occurring worldwide and a lack of accurate death registration systems is common in most developing countries, particularly where mortality is highest [3].

As in other Sub-Sahara African countries, in Angola the causes of death and their determinants are not well documented [3–5]. In recent years, Angola faced great social and economic changes, resulting from the end of an armed conflict of almost 30 years. Furthermore, in less than a decade, a vibrant economy has transformed the country from a low income centrally-planned system to a middle income market economy [6, 7]. Such development brings changes in the societal and economic structure which, in turn, impacts health and mortality patterns [8–10]. However, the resources needed to implement an accurate and complete vital statistics system are not yet fulfilled.

Even though World Health Organization (WHO) provides estimates of deaths by cause for the country (the main causes in 2012 were respiratory infections, diarrhea, neonatal deaths, cardiovascular diseases and malaria [11]), these figures pertain to aggregated data, not describing regional patterns (for example, cause-specific mortality in identified malaria endemic areas). In Angola, death certification is only done routinely for violent deaths and on those occurring in hospitals or in other health facilities [12]. For example, it is estimated that only about 5 % of under-five children deaths take place at the hospitals [13] and that about 45 % of the population has access to any type of health care [14]. The system thus fails to collect data on deaths occurring at home or at small health units and is therefore insufficient to reliably inform about mortality.

In countries with limited or non-existent death registration systems, the use of verbal autopsy (VA) is recommended to ascertain probable causes of death [1]. Verbal autopsy as a method to estimate cause-specific mortality, but also to study risk factors for specific diseases and the effects of public health interventions, is increasing in the developing world and is now used in more than 115 countries [1, 3]. Verbal autopsy systems are often developed as part of population monitoring platforms, namely Health and Demographic Surveillance Systems (HDSS), to allow longitudinal assessment of mortality trends and exploration of associated factors. The Dande HDSS (Bengo, northern Angola), where a VA system is implemented since 2010, provides such an opportunity [15].

This study aims to describe the main causes of deaths and explore factors associated with broad mortality groups for the period 2009–2012 in the Dande HDSS study area.

## Methods

### Study area and population

The Dande HDSS is located in Dande municipality, Bengo province, about 60 km northeast of Luanda, in Northen Angola. It was established and is managed by CISA—Health Research Centre in Angola—to overcome the scarcity of complete vital records, the lack of knowledge on demographic characteristics and living conditions of the population in this region and to create a sampling frame for epidemiological studies. Detailed information about the HDSS scope, design and implementation, methodology and data management procedures is published elsewhere [15].

**Fig. 1:**
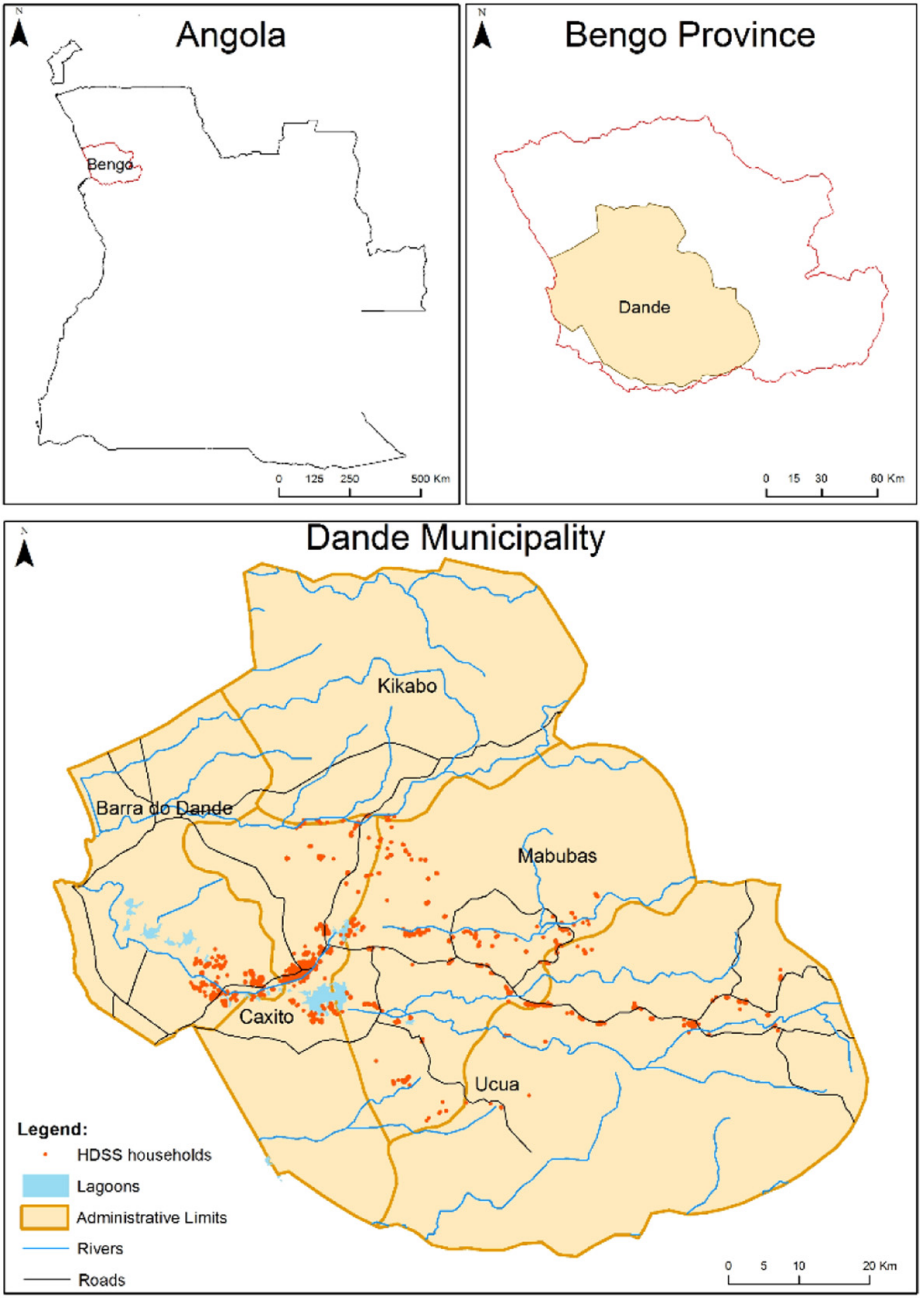
Map of the study area—Angola, Bengo Province, Dande Municipality

The Dande HDSS covers all 71 hamlets of Caxito, Mabubas and Úcua, 3 of the 6 communes composing the Dande municipality, with a contiguous total area of 4763.6 km^2^ (Fig. 1).

Thirty hamlets are considered urban (agglomerations of 2000 or more inhabitants and basic infrastructures) and 41 are rural, according to the National Institute of Statistics of Angola (INEA) definitions [16]. In the initial census, performed in 2009, a population of 59,683 individuals living in 15,643 households was registered. In December 2012, the population covered by the HDSS was of 60,614 residents.

The Angolan Health System encompasses public and private sector providers, the former being the main provider. The national public health system is hierarchically organized in primary health care centers (these include small health care units, nursing centres and municipal hospitals), secondary level general hospitals and tertiary level reference hospitals [14]. The Dande Municipality, where the study area is located, is served by eleven primary health care facilities, one health centre, one maternal and infant centre, one municipal hospital and one general hospital [17].

### Data collection

After the initial census, that was performed between August 2009 and March 2010, 18 fieldworkers periodically visited all the households of the study area to record basic demographic events, including reported deaths (quarterly update rounds took place until the end of 2011 and, due to logistic and administrative issues, a single 6-month round was performed in 2012). This study covers the deaths which occurred from August 2009 to December 2012.

After a mourning period of approximately a month and a half, three fieldworkers specifically trained to apply the VA tool, individually visited the households of the deceased and interviewed the caregiver of the deceased or other close relative.

Three versions of the standard INDEPTH/WHO model of the VA questionnaire [18, 19] were used (one for neonatal deaths—0 to 27 days, one for children—28 days to 14 years, and one for adults—15 years or more) adapted to include the main pathologies known in the region and local terminologies. The VA questionnaires are structured into different sections, and include open questions on the circumstances of the death, sociodemographic data of the interviewee and of the deceased, a checklist of signs and symptoms experienced before death (including their duration), accidents, accessibility to health care, place of death and any available health records, namely vaccination register, laboratory exams results and death certificate.

### Assigning the cause of death

To ascertain the probable cause of death, each questionnaire was interpreted independently by two physicians with local experience and trained on reviewing the VA questionnaires and assigning the cause of death. Training of physicians in the interpretation of the questionnaires was based on case definition using clinical diagnostic algorithms adapted from other VA systems [18, 20–23]. In case of discordance between the two physicians, the questionnaire was reviewed by a third physician and the majority rule was applied. Otherwise, if the third physician determined a different cause of death, the case was registered as indeterminate [24, 25].

The reading physicians were staff of the General Provincial Hospital, partner of the Health Research Centre of Angola and also the setting where the Centre is located. This clinical staff is mostly composed of Angolan national practitioners, although there are also foreign doctors (Cuban) working in this context for several years, as part of bilateral cooperation agreements between governments. For each VA questionnaire reading, the Centre paid a symbolic fee to the physicians, in order to compensate their participation. The Hospital benefits of the results of the verbal autopsy system, an important tool to the knowledge of the main causes of death in the study area.

The international form for medical certificate of cause of death was used, which is divided in two parts: the first describes the sequence of morbid conditions directly leading to death; the second describes other diseases or conditions which may have contributed to the death, but which were not involved in the fatal sequence.

Though the international death certificate typically uses the underlying cause of death, ascertaining a single cause of death from various possible causes identifiable from the reported symptoms may be inappropriate using VA data [1]. Mortality is often due to the effects of multiple conditions, and particularly in the case of infant death, it is more difficult to distinguish, in terms of semiology and differential diagnosis, the main causes that lead to death. Additionally, concerning adults is quite straightforward the distinction between comorbidities and underlying cause for death. For those reasons, multiple causes of death were considered for children under 15 years old and the underlying cause of death was considered for adults.

Causes of death were coded using the 10^th^ revision of the International Classification of Diseases (ICD-10) [26].

### Categorization of causes of death

After classification based on ICD-10, specific causes of death were categorized according to the three mortality groups of the Global Burden of Disease (GBD) structure [27] and a fourth for indeterminate causes.

Group I included deaths attributed to communicable diseases and maternal, perinatal and nutritional conditions (CD): intestinal infectious diseases, tuberculosis (TB), other bacterial diseases, rabies, measles, viral hepatitis, human immunodeficiency virus (HIV), malaria, nutritional deficiencies, meningitis, otitis, conditions related to or aggravated by the pregnancy, childbirth or by the puerperium (maternal causes or obstetric causes) and certain conditions originating in the perinatal period.

Group II consisted of non-communicable diseases (NCD): malignant neoplasms, respiratory disorders, mental and behavioral disorders, epilepsy, diseases of the circulatory system, diseases of the digestive system, diseases of the genitourinary system, skin disorders and congenital abnormalities.

Group III comprised of injuries (INJ): injury, poisoning and other consequences of external causes of morbidity and mortality, such as burns and corrosions, toxic effects of substances chiefly nonmedicinal as to source, complications of surgical and medical care, traffic accidents, intentional self-harm, assault, event of undetermined intent, accidental drowning and submersion, struck by thrown, projected or falling object, falls, contact with scorpions, exposure to unspecific electric current and handgun discharge.

Indeterminate causes (IND) included: unknown and unspecified causes of mortality (physicians assigned the VA as ill-defined or as unknown cause of mortality—codes R95-R99 of ICD-10) and those cases that the VA method didn’t reach a consensus on the cause of death.

### Demographic and socioeconomic characteristics

During the initial census of the HDSS, implemented from August 2009 to March 2010, and during the 5^th^ update round that took place from September to December 2011, demographic characteristics (age and sex) and information on household conditions were collected. In each HDSS update round, demographic events are updated and all the information is collected for new members in the study area.

The educational level of the deceased (or the mother’s educational level for children under 15 years of age) was categorized in three groups: no formal education; from one to four—corresponding to primary education; and five or more years of completed schooling.

Several household conditions were used to create a proxy socioeconomic position (SEP) index: type of walls (made of clay, wattle and daub, bricks), type of roof (straw, tin, tile, other), main source of drinking water (public fountain, piped water, river, irrigation ditch, well, plumbed, public tank) and existence of a latrine (with or without water and private or shared with neighbors). For each identified death the most recently updated information on their household conditions was used. The place of residence of the deceased (dichotomized in rural or urban) and place of death (in a health facility or in other/unknown location), were also used.

### Statistical analysis

Proportions of the main causes of deaths were computed by sex and the six age groups most commonly used to describe age-specific mortality (<28 days, from 28 days to 12 months, 1 to 5 years, 5 to 15 years, 15 to 50 years and 50 or more years of age).

The chi-square test was used to compare proportions of deaths with and without an assigned cause according to sex and age (Additional file 1).

Principal component analysis (PCA) was used to summarize household conditions and create the SEP index. The first principal component extracted, which explained 17.8 % of variance was then categorized into SEP quintiles (lowest, low, medium, high and highest).

Proportions of the grouped causes of death (CD, NCD, INJ and IND), according to sex, age (for the bivariate and multivariate analysis, age was re-categorized in four groups: under 5 years, 5 to 14, 15–49 and 50 or more), educational level, the quintiles of the SEP index, place of residence (rural or urban), and place of death (in a health facility or other/unknown) were compared using the chi-square test.

The WHO standard population (2000–2025) [28] was used to compute standardized deaths rates using the direct method.

Fully adjusted multivariate logistic regression models were fitted and odds ratios (95 % confidence intervals - OR, 95 % CI) computed to estimate associations between each main group of cause of death (CD, NCD, INJ and IND) and all demographic and socioeconomic characteristics considered in the bivariate analysis. Models were additionally adjusted for multiple causes of death by adding a dichotomized dummy variable signaling multiple cause cases. Only participants with complete information were used in the regression models. Two variables presented missing information, namely education (5.5 % of missing values) and socio economic position index (0.1 % missing). Analysis was performed using the software SPSS v.22.

### Ethical consideration

In the Dande HDSS, we sought verbal consent from household heads or any other adult member of the household, to update the health and demographic information of household members every 6 months. Verbal autopsies for the reported deaths were included as operations of the Dande HDSS.

As a health and demographic surveillance system with frequent visits to households to update demographic information, verbal consent was deemed appropriate for monitoring of the demographic events. In the case of VA, informed written consent (signed or fingerprinted in case of illiteracy) was required before the conduct of each interview. The information given out to respondents during the consent process was documented and fieldworkers have been adequately trained to administer the consent. The consent information included the aim of the verbal autopsy system, the content of the participation of the caregiver (to answer to an interview, giving information about the death of her/his relative), the voluntary feature of participation and the confidentiality of the information. Consent forms were kept safe, strictly confidential and separated from questionnaires.

The scientific committee of CISA, composed of several biomedical scientists, epidemiology and public health experts acted as an institutional review board (IRB) for all studies and reviewed the procedures of the HDSS and VA systems. The IRB approved the consent process and also asked for the advice of the Ministry of Health and of Provincial Health Governor. According to their recommendations, the forms of the HDSS and VA were approved and registered in the National Statistics Institute of Angola (INEA).

The STROBE guidelines were followed.

## Results

From August 2009 to December 2012 a total of 1488 deaths were registered by the Dande HDSS, 811 (54.5 %) were males. The mean age at death was 30.0 years (standard deviation = 28.1), with 513 (34.5 %) occurring in children under 5 years of age.

The crude mortality rate in 2010 was 8.7 per 1000 inhabitants, (95 % confidence interval CI: 8.0–9.5) and 8.1 per 1000 (95 % CI: 7.4–8.8) in 2011. In 2012, the estimated crude mortality rate was 6.6 (95 % CI: 5.9–7.2). The standardized deaths rates (WHO standard population 2000–2025) were 10.8, 9.8 and 9.3 per 1000 for 2010, 2011 and 2012 respectively.

Infant mortality rate was 67.1 deaths per 1000 live births in 2010, 78.4 in 2011, and 60.2 in 2012. Under five mortality rate was 90.9, 93.6 and 71.5 per 1000 live births in 2010, 2011 and 2012 respectively.

Of the 1488 deaths, 1009 (67.8 %) verbal autopsies were made. Five families refused (0.3 %) to participate in the study. The difference between the number of reported deaths and the number of interviews was mainly due to 316 families that migrated out of or in the study area (21.2 %), 27 individuals who lived alone at the time of death (1.8 %) making it very difficult to find a primary caregiver to interview and 131 families who were not at home in the various attempts done to conduct the interview (8.8 %). The periodic visits of the HDSS to households allows the surveillance of the population, however when individuals migrate within the study area they are registered in the new house only during the next update round, making it difficult to locate them in the periods between the visits.

Of the 1009 completed verbal autopsies 798 were reviewed and a cause of death was attributed. Of the total of 798 with assigned causes of death, 458 (57 %) had to be reviewed by a third doctor. The remaining 211 were not included in the analysis because they were not read by at least two physicians and therefore had no cause of death assigned. When considering multiple causes of death in children, a total of 934 causes of death were assigned and included in the current analysis. A statistically significant difference in the proportion of deaths with and without a VA reviewed was observed considering the six-age group categories, and no sex-difference was found (Additional file 1: Table S1).

This study revealed that malaria is the most common cause of death in the Dande HDSS, Angola, for the period August 2009 to December 2012, affecting mostly children and adolescents less than 15 years of age. Intestinal infectious diseases, malnutrition and pneumonia were also main contributors to child mortality. For adults, the most common causes of death are diseases of the circulatory system, TB, traffic accidents and malaria.

### Specific cause of death by age group and sex

#### Neonatal deaths

All deaths in female infants <28 days and for 47.4 % of males, were classified as having a perinatal condition as cause of death (Table 1).

**Table 1:** Causes of neonatal death (children < 28 days)

< 28 days	Female (*n* = 8)		Male (*n* = 19)		Total (*N* = 27)	
	N	%	n	%	N	%
Perinatal period conditions	8	100.0	9	47.4	17	63.0
Intestinal infectious diseases (diarrhea)	0	0.0	1	5.3	1	3.7
Malaria	0	0.0	1	5.3	1	3.7
Meningitis	0	0.0	1	5.3	1	3.7
Traffic accidents	0	0.0	1	5.3	1	3.7
Unknow (R95-R99)	0	0.0	1	5.3	1	3.7
Indeterminate	0	0.0	5	26.3	5	18.5
Total	8		19		27	

#### Children

In children from 28 days to 11 months of age (Table 2), the main causes of death were intestinal infectious diseases (female: 26.7 %; male: 34.4 %) and malaria (female: 19.8 %; male: 26.7 %). Those were followed by pneumonia (female: 18.6 %; male: 14.4 %) and malnutrition (female: 15.1 %; male: 11.1 %).

**Table 2:** Causes of death among infants from 28 days to 11 months

28 days-11 months	Female (*n* = 61)		Male (*n* = 63)		Total (*N* = 124)	
	N	%	n	%	N	%
Intestinal infectious diseases (diarrhea)	23	26.7	31	34.4	54	30.7
Malaria	17	19.8	24	26.7	41	23.3
Acute respiratory infections (pneumonia)	16	18.6	13	14.4	29	16.5
Malnutrition	13	15.1	10	11.1	23	13.1
Meningitis	4	4.7	1	1.1	5	2.8
Tuberculosis	3	3.5	0	0.0	3	1.7
Anaemia	3	3.5	0	0.0	3	1.7
Measles	2	2.3	0	0.0	2	1.1
Hepatitis	0	0.0	1	1.1	1	0.6
Lower respiratory infections	0	0.0	1	1.1	1	0.6
Digestive system	0	0.0	1	1.1	1	0.6
Perinatal period	1	1.2	0	0.0	1	0.6
Burn and corrosions	0	0.0	1	1.1	1	0.6
Accidental drowning and submersion	0	0.0	1	1.1	1	0.6
Indeterminate	4	4.7	6	6.7	10	5.7
Total (multiple causes considered)	86		90		176	

The same diseases were the main causes of deaths among children aged 1–4 years (Table 3): malaria (female: 32.8 %; male: 35.4 %), intestinal infectious diseases (female: 17.6 %; male: 22.0 %), malnutrition (female: 16.0 %; male: 13.4 %) and pneumonia (female: 13.0 %; male: 6.3 %).

**Table 3:** Causes of death among children from 1 to 4 years of age

1–4 years	Female (*n* = 87)		Male (*n* = 95)		Total (*N* = 182)	
	N	%	N	%	N	%
Malaria	43	32.8	45	35.4	88	34.1
Intestinal infectious diseases (diarrhea)	23	17.6	28	22.0	51	19.8
Malnutrition	21	16.0	17	13.4	38	14.7
Acute respiratory infections (pneumonia)	17	13.0	8	6.3	25	9.7
Measles	5	3.8	7	5.5	12	4.6
Accidental drowning and submersion	2	1.5	5	3.9	7	2.7
Burn and corrosion	2	1.5	1	0.8	3	1.2
Infections of the skin	1	0.8	2	1.6	3	1.2
Meningitis	2	1.5	0	0.0	2	0.8
Congenital malform. circulatory system	2	1.5	0	0.0	2	0.8
Sepsis	1	0.8	1	0.8	2	0.8
HIV	1	0.8	1	0.8	2	0.8
Anaemia	0	0.0	2	1.6	2	0.8
Traffic accidents	1	0.8	0	0.0	1	0.4
Falls	1	0.8	0	0.0	1	0.4
Struck by thrown. proj. or falling object	1	0.8	0	0.0	1	0.4
Digestive system	0	0.0	1	0.8	1	0.4
Nephrotic syndrome	0	0.0	1	0.8	1	0.4
Unknow (R95-R99)	2	1.5	1	0.8	3	1.2
Indeterminate	6	4.6	7	5.5	13	5.0
Total (multiple causes considered)	131		127		258	

Malaria was also the leading cause of death among 5–14 years old children (Table 4), (female: 28.1 %; male: 38.2 %) followed by intestinal infectious diseases (female: 18.8 %; male: 17.6 %). The deaths due to accidental drowning and submersion (female: 3.1 %; male: 14.1 %) were the third specific cause of death for this age group.

**Table 4:** Causes of death among children from 5 to 14 years of age

5–14 years	Female (*n* = 27)		Male (*n* = 31)		Total (*N* = 58)	
	N	%	N	%	N	%
Malaria	9	28.1	13	38.2	22	38.6
Intestinal infectious diseases (diarrhea)	6	18.8	6	17.6	12	18.2
Accidental drowning and submersion	1	3.1	5	14.7	6	9.1
Malnutrition	1	3.1	2	5.9	3	4.5
Acute respiratory infections (pneumonia)	2	6.3	0	0.0	2	3.0
Traffic accidents	2	6.3	0	0.0	2	3.0
Tuberculosis	0	0.0	1	2.9	1	1.5
Sepsis	1	3.1	0	0.0	1	1.5
Rabies	1	3.1	0	0.0	1	1.5
Otitis	0	0.0	1	2.9	1	1.5
Injuries knee and lower leg	1	3.1	0	0.0	1	1.5
Toxic effects of nonmedicinal substances	0	0.0	1	2.9	1	1.5
Falls	1	3.1	0	0.0	1	1.5
Exposure to unspecified electric current	0	0.0	1	2.9	1	1.5
Meningitis	1	3.1	0	0.0	1	1.5
Malign neoplasms	1	3.1	0	0.0	1	1.5
Unknow (R95-R99)	1	3.1	2	5.9	3	4.5
Indeterminate	4	12.5	2	5.9	6	9.1
Total (multiple causes considered)	32		34		66	

#### Adults

In adults (Table 5), a quarter of all deaths were classified as indeterminate (25.0 %). In the age group 15–49 years, 11.3 % of women died of causes related with pregnancy, childbirth and puerperium, followed by malaria and diseases of the circulatory system (both with 7.5 %). The main causes for male death were TB (15.8 %), traffic accidents (13.3 %) and malaria (8.3 %).

**Table 5:** Causes of death among adults aged 15–49 years

15–49 years	Female		Male		Total	
	n	%	N	%	N	%
Tuberculosis	8	10.0	19	15.8	27	13.5
Traffic accidents	5	6.3	16	13.3	21	10.5
Malaria	6	7.5	10	8.3	16	8.0
Circulatory system diseases	6	7.5	5	4.2	11	5.5
Digestive system	5	6.3	4	3.3	9	4.5
Preg. childbirth and puerperium	9	11.3	0	0.0	9	4.5
Intestinal infectious diseases (diarrhea)	3	3.8	4	3.3	7	3.5
HIV	4	5.0	3	2.5	7	3.5
Accidental drowning and sub.	3	3.8	4	3.3	7	3.5
Intentional self-harm	0	0.0	4	3.3	4	2.0
Burn and corrosion	2	2.5	1	0.8	3	1.5
Malignant neoplasms	0	0.0	2	1.7	2	1.0
Epilepsy	0	0.0	2	1.7	2	1.0
Acute respiratory infections (pneumonia)	0	0.0	2	1.7	2	1.0
Unsp. electric current exposure	0	0.0	2	1.7	2	1.0
Rabies	1	1.3	0	0.0	1	0.5
Acute upper respir. Infections	0	0.0	1	0.8	1	0.5
Conditions of lower respiratory tract	0	0.0	1	0.8	1	0.5
Infections of the skin and subcut. Tissue	0	0.0	1	0.8	1	0.5
Toxic effects of nonmedicinal substances	1	1.3	0	0.0	1	0.5
Complications of surgical and medical care	1	1.3	0	0.0	1	0.5
Handgun discharge	0	0.0	1	0.8	1	0.5
Contact with scorpions	0	0.0	1	0.8	1	0.5
Assault	0	0.0	1	0.8	1	0.5
Event of undetermined intent	0	0.0	1	0.8	1	0.5
Unknow (R95-R99)	5	6.3	6	5.0	11	5.5
Indeterminate	21	26.3	29	24.2	50	25.0
Total	80		120		200	

From age 50 onwards (Table 6), 30.0 % of all deaths had indeterminate causes of death. Diseases of the circulatory system were the most common cause of death identified for both sexes (female: 27.6 %; male: 18.6 %), followed by TB (female: 6.7 %; male: 9.8 %) and malaria (female: 7.6 %; male: 7.8 %).

**Table 6:** Causes of death among adults aged 50 or more years

50 years +	Female		Male		Total	
	N	%	n	%	N	%
Circulatory system diseases	29	27.6	19	18.6	48	23.2
Tuberculosis	7	6.7	10	9.8	17	8.2
Malaria	8	7.6	8	7.8	16	7.7
Intestinal infectious diseases (diarrhea)	3	2.9	6	5.9	9	4.3
Malignant neoplasms	5	4.8	3	2.9	8	3.9
Traffic accidents	2	1.9	5	4.9	7	3.4
Acute respiratory infections (pneumonia)	4	3.8	3	2.9	7	3.4
HIV	4	3.8	1	1.0	5	2.4
Diabetes Mellitus	1	1.0	4	3.9	5	2.4
Digestive system	1	1.0	3	2.9	4	1.9
Intentional self-harm	2	1.9	1	1.0	3	1.4
Accidental drowning and sub.	0	0.0	2	2.0	2	1.0
Rabies	1	1.0	0	0.0	1	0.5
Mental and behavioural disorders	0	0.0	1	1.0	1	0.5
Infections of the skin and subcutaneous tissue	0	0.0	1	1.0	1	0.5
Injury of unspecified body region	1	1.0	0	0.0	1	0.5
Exposure to unspecified electric current	1	1.0	0	0.0	1	0.5
Sepsis	0	0.0	1	1.0	1	0.5
Asthma	0	0.0	1	1.0	1	0.5
Unknow (R95-R99)	1	1.0	6	5.9	7	3.4
Indeterminate	35	33.3	27	26.5	62	30.0
Total	105		102		207	

#### Main categories of mortality according to demographic characteristics, socioeconomic indicators, place of residence and place of death

When specific causes of death are aggregated into GBD main categories of mortality, CD are the leading cause of deaths (61.0 %), followed by NCD (11.6 %) and INJ (9.1 %). Within CD deaths, 11.2 % were attributed to nutritional conditions, 3.2 % to perinatal conditions and 1.6 % to maternal causes (Additional file 1: Table S2).

A total of 18.3 % of the causes of death were IND (physicians assigned the VA as ill-defined or the three different physicians didn’t reach a consensus on the suggested cause of death) (Table 7).

**Table 7:** Distribution of the main causes of death according to demographic and socioeconomic characteristics

		Total	Communicable	Non-Communicable	Injuries	Indeterminate	
		n	n (%)	n (%)	n (%)	n (%)	p
Sex	Female	442	278 (48.8)	55 (50.9)	30 (35.3)	79 (46.2)	0.109
	Male	492	292 (51.2)	53 (49.1)	55 (64.7)	92 (53.8)	
Age (years)	< 5	461	399 (70.0)	14 (13.0)	16 (18.8)	32 (18.7)	<0.001
	5–14	66	44 (7.7)	1 (0.9)	12 (14.1)	9 (5.3)	
	15–49	200	71 (12.5)	25 (23.1)	43 (50.6)	61 (35.7)	
	50+	207	56 (9.8)	68 (63.0)	14 (16.5)	69 (40.4)	
Education^a^ (years)	None	332	202 (38.1)	52 (49.1)	16 (20.3)	62 (36.9)	0.001
	1 to 4	316	202 (38.1)	27 (25.5)	31 (39.2)	56 (33.3)	
	5 or more	235	126 (23.8)	27 (25.5)	32 (40.5)	50 (29.8)	
SEP index	Lowest	186	121 (21.2)	23 (21.3)	12 (14.1)	30 (17.6)	0.053
	Low	222	143 (25.1)	27 (25.0)	13 (15.3)	39 (22.9)	
	Medium	170	108 (18.9)	14 (13.0)	21 (24.7)	27 (15.9)	
	High	171	107 (18.8)	18 (16.7)	16 (18.7)	30 (17.6)	
	Highest	184	91 (16.0)	26 (24.1)	23 (27.1)	44 (25.9)	
Residence	Rural	180	88 (15.4)	23 (21.3)	32 (37.6)	37 (21.6)	<0.001
	Urban	754	482 (84.6)	85 (78.7)	53 (62.4)	134 (78.4)	
Place of death	Health facility	490	338 (59.3)	59 (54.6)	22 (25.9)	71 (41.5)	<0.001
	Other/unknown	444	232 (40.7)	49 (45.4)	63 (74.1)	100 (58.5)	

*p* p-value—chi square test

SEP index: socioeconomic position index

^a^For participants under 15 years of age, the mother’s educational level was considered

Statistically significant differences were found across all groups for age, education, living in an urban or rural setting and place of death. Deaths in all categories were proportionally more frequent in males than in females except for NCD, although no statistically significant sex-difference was found. Deaths from CD were more frequent in children under 5 years of age (causing 70.0 % of deaths in this age group) while those due to NCD were more frequent in people older than 50 years (63.0 %). INJ were more frequent in the age group 15–49 years (causing 50.6 % of deaths in this age group) and IND causes of death among adults 50 or more years (40.4 %). Mortality was highest among those with no formal education, except for the group of deaths attributed to INJ where people with 5 or more years of school died more frequently (40.5 %). There was no significant relation between the SEP index and the main groups of causes of death (*p* = 0.053). The majority of deaths with an attributed cause occurred in individuals living in urban areas (*p* = <0.001).

Deaths due to CD or NCD were more frequent at health facilities (CD: 59.3 % and NCD: 54.6 %) and deaths caused by INJ and IND occurred mostly on other/unknown place (INJ: 74.1 %, IND: 58.5 %, respectively).

In general, the bivariate associations described above were confirmed in the fully adjusted regression models (Table 8). Children under five were more likely to die from CD when compared to adults aged 50 or more years (adjusted OR, 95 % CI: 8.45, 5.33–13.41), and less likely to die from NCD (aOR 0.09 95 % CI 0.04–0.18). The odds of dying of injuries were 3.91 times (95 % CI: 1.33–11.48) higher for children aged 5–14 years compared to adults older than 50 years and the number of IND causes of death tended to increase with age, independently of the remaining factors.

**Table 8:** Associations between causes of death and socioeconomic characteristics

		Communicable	Non- Communicable	Injuries	Indeterminate
		*AOR (95 % CI)	*AOR (95 % CI)	*AOR (95 % CI)	*AOR (95 % CI)
Sex	Male	1	1	1	1
	Female	0.92 (0.64–1.32)	1.11 (0.67–1.82)	0.77 (0.44–1.37)	1.18 (0.79–1.76)
Age (years)	<5	8.45 (5.33–13.41)	0.09 (0.04–0.18)	1.01 (0.45–2.26)	0.33 (0.20–0.55)
	5–14	5.36 (2.49–11.54)	n.a.	3.91 (1.33–11.48)	0.38 (0.15–0.97)
	15–49	1.67 (1.05–2.64)	0.29 (0.16–0.50)	3.60 (1.78–7.29)	0.85 (0.54–1.33)
	50+	1	1	1	1
Education^a^ (years)	None	1.68 (1.04–2.72)	1.08 (0.57–2.07)	0.46 (0.21–0.98)	0.77 (0.46–1.30)
	1–4	1.15 (0.74–1.79)	0.81 (0.44–1.51)	0.93 (0.50–1.75)	1.03 (0.64–1.65)
	5 or more	1	1	1	1
SEP index	Lowest	1.16 (0.66–2.05)	1.04 (0.50–2.16)	1.18 (0.48–2.92)	0.73 (0.40–1.34)
	Low	1.21 (0.71–2.05)	0.96 (0.49–1.88)	0.86 (0.37–1.98)	0.84 (0.49–1.46)
	Med	0.84 (0.47–1.50)	0.80 (0.36–1.77)	2.31 (1.00–5.30)	0.84 (0.46–1.56)
	High	0.81 (0.46–1.44)	0.96 (0.46–2.01)	1.43 (0.63–3.28)	1.03 (0.57–1.86)
	Highest	1	1	1	1
Residence	Rural	0.65 (0.41–1.05)	0.86 (0.47–1.60)	3.23 (1.71–6.10)	0.87 (0.53–1.42)
	Urban	1	1	1	1
Place of death	Health facility	1	1	1	1
	Other/unknown	0.56 (0.40–0.79)	0.59 (0.37–0.93)	3.57 (2.04–6.25)	1.44 (0.99–2.08)

*AOR (95 % CI) = adjusted odds ratio (95 % confidence Interval). adjusted for all variables listed and for multiple causes

SEP index: socioeconomic position index

^a^For participants under 15 years of age, the mother’s educational level was considered

**Fig. 2:**
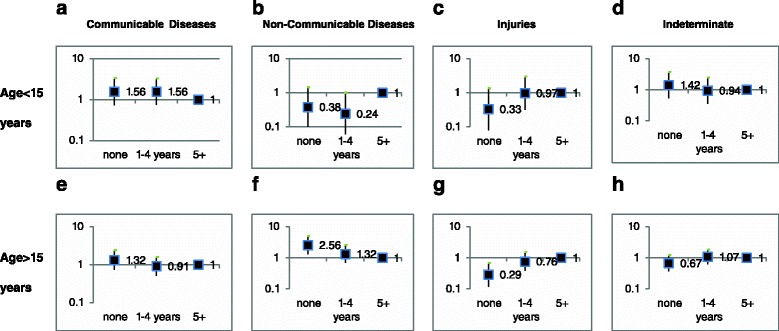
Associations (adjusted Odds Ratio, OR, 95% Confidence Intervals, CI) between Educational level (none, 1-4 years, 5 or more years of schooling) and main causes of death among those under 15 years of age and among those aged 15 or more for deaths caused by Communicable Diseases (**a**), Non-Communicable Diseases (**b**), Injuries (**c**), and Indeterminate (**d**). Marks represent OR estimates and bars represent 95% CI. Models for under-15 were adjusted for sex, place of residence, local of death and multiple causes and models for 15 years or more were adjusted for sex, place of residence and local of death

Compared to individuals with 5 or more years of education, those with no formal education were more likely to die of CD causes (aOR 1.68 95 % CI 1.04–2.72) and less likely to die of INJ (aOR 0.46 95 % 0.21–0.98), independently of age, sex, place of residence and place of death. When stratified by age (under 15 years of age or above), adults with no formal education were significantly more likely to die of NCD causes (aOR 2.56 95 % CI 1.30–5.02) and significantly less likely to die of INJ (aOR 0.29 95 % CI 0.12–0.67) compared to those with 5 or more years of education (Fig. 2f and g, respectively).

Compared to those in the highest quintile of the SEP index, individuals in the medium SEP index quintile were more likely to die from INJ (aOR 2.31 95 % CI 1.00–5.30), with no other statistically significant association found for the remaining SEP groups.

Living in a rural setting represented 3.23 (95 % CI: 1.71–6.10) higher odds of dying of INJ compared to residents in urban areas.

Deaths from CD and NCD, were more frequently observed in a health facility (aOR 0.56 95 % CI0.40–0.79 and 0.59, 0.37–0.93, respectively) and deaths from INJ were, expectedly, more frequent in other/unknown places (aOR 3.57 95 % CI 2.04–6.25).

## Discussion

The high proportion of deaths occurring in children under five (33.9 % of all deaths occur in this age group) is characteristic of a developing country [4, 29, 30] and is consistent with the WHO latest mortality data estimates for Angola [11]. The 2012 WHO estimates for the distribution of causes of death in children under 5, place respiratory diseases (17.1 %), diarrhea (14.6 %) and malaria (12.6 %) as the main causes of death, which is similar to our findings, although with a higher burden for deaths caused by malaria (28.2 %), diarrhea (23.0 %) and a lower frequency of respiratory diseases as causes of death (11.9 %). Being the Dande HDSS study area a malaria endemic region [31, 32], there might be a tendency to over diagnose malaria and misclassify other fever deaths [33–35]. Over diagnosis of a particular cause of death, known as endemic in a particular region, has been pointed as one of the limitations of the VA methodology, reducing its performance [1, 4, 35]. One other factor that may have contributed to an overestimation of malaria and intestinal infections as causes of death, was the methodology we opted for assigning multiple causes for neonatal and children deaths. Multiple effects and several medical conditions influence mortality, particularly in children and the elderly [36], and in contexts like Dande, the selection of the underlying cause of death is often arbitrary [4, 37]. One of the arguments commonly used in favor of the methodology which involves assigning multiple causes of death is related with the lack of criteria to select a single cause of death to the detriment of others. If the VA system aims to understand epidemiological needs at a population level, rather than describe the causes of deaths at individual level, selecting a single cause may be less appropriate than assigning multiple causes, particularly for the purposes of public-health support [1].

The most common causes of death among all adults aged 15–49 years old were TB and traffic accidents, although for women, pregnancy, childbirth and puerperium complications were responsible for the highest proportion of deaths. The National Plan for Development of Health identifies TB and traffic accidents as enormous problems for the population aged 15–45 years old [14], although accurate statistics are not provided. The Global Health Estimates [11], point to a lower proportion of TB and a higher burden of HIV deaths in this age group. As reported in other studies [4, 29], VA might have contributed to an underestimation of HIV-related deaths, since the co-morbidity of TB and HIV [38, 39] might have influenced the weight of both diagnoses. Traffic accidents were recently pointed as the second major cause of death in the country by the government in the media [40]. The increased availability of motor vehicles and the improvement in life conditions is likely to raise the burden of traffic INJ in this age group [41], which adds to the poor road infrastructures and drinking and driving culture.

Regarding deaths among women of reproductive age, our results confirm the need to prioritize interventions to improve reproductive and maternal health, since maternal-related deaths rates in Angola are amongst the highest in the world [42], and the reduction of its burden is a specific goal envisaged in the Millennium Development Goals.

Among the eldest groups (50 or more years of age), circulatory system diseases were the leading causes of death. Such finding is consistent with the results from other studies showing that deaths related to the circulatory system, particularly cardiovascular diseases, increases with age [5, 29, 33, 43]. In 2010, our group conducted a community-based survey of 1464 adults residing in the Dande municipality, and found a 23 % prevalence of hypertension [44]. These findings suggest an expected increase in the burden attributable to chronic diseases among adults, resultant from the country’s fast economic growth and lifestyles changes.

Overall, our results show similar distributions of deaths by broad GBD groups compared to those found in Tanzania [33], particularly in what concerns NCD (12 % in our study vs. 15 % in Tanzania) and INJ (9 % in both studies). The higher estimate of CD found in our study (61 %) might be explained by the inclusion of children (the Tanzania study, focused on adult deaths, and found 41 % of CD deaths) and is more close to the findings of a study among adolescents and adults conducted in rural western Kenya, which found 74 % of deaths caused by CD [29].

Mortality in these regions is still largely marked by infectious diseases, despite the increasing deaths caused by NCD and INJ. This phenomenon, which a majority of developing countries in Africa is facing, has been called the “triple burden” [29, 45]. Some of the factors usually linked to the increasing prevalence of NCD and INJ in developing countries are related with socioeconomic conditions improvement which impacts lifestyles changes. Therefore we explored the associations of socioeconomic conditions in broad mortality groups. For this, we used essentially two SEP proxy measures: the educational level and the quintiles of an index summarizing household conditions, which has been extensively used in other HDSSs to study inequalities in several health outcomes [8, 25, 29, 33, 46–48].

No significant associations were observed according to the quintiles of the SEP index created, although point estimates suggest positive associations in those groups who might be considered socially disadvantaged, compared to those less disadvantaged, for CD and NCD. The gradient observed in CD deaths according to the educational level, is in line with the results of a recent study conducted in Chakaria, Bangladesh, where mortality due to CD was more frequent among the poorest [46]. In the same study, NCD as leading causes of death also concentrated among the population from the higher SEP groups [47], contrasting our results for adult NCD deaths according to the educational level as shown in Fig. 2f. This may suggest that the expected epidemiological transition in the leading causes of death is only in its early stage [49, 50].

Nevertheless, we would expect a steeper socioeconomic gradient for all main causes of death, given the fast development observed in the country in the last decade [6] and the known inequalities in mortality observed worldwide and in the country [42]. It would be interesting to explore other SEP measures, such as the subjective social status [51], potentially more useful to disentangle underlying inequalities of health in this context.

Most deaths studied occurred in the urban area, following the population distribution in the study area. The higher likelihood of an INJ death in residents from rural compared to urban hamlets was a rather surprising finding. However, we should notice that only the deceased residence is known, and death might have happened in an urban area (outside a health facility). When isolating the specific causes of death by injuries, we can observe the weight of those related with traffic accidents and accidental drowning and submersion, both in rural and urban areas. In rural areas the villages are situated along the road and those have relatively poor conditions and some traffic, especially of trucks transporting raw materials. This has been the cause of road and pedestrian accidents. Likewise, people living in rural areas have to travel long distances which may explain the higher likelihood to die of INJ, particularly of transport accidents.

To better understand the context of INJ deaths, we recently included a specific module in our VA questionnaire, which will be the focus of future exploration (as the characterization and context of the main deaths caused by INJ in order to sustain public health interventions and specific campaigns).

Regarding place of death, CD and NCD deaths happened more commonly in a health facility. Nevertheless, more than 40 % of CD and NCD deaths were registered outside a health facility, which emphasizes the usefulness of the VA system in the region.

The identification of causes of deaths through VA methodology in the scope of a continuous surveillance system is the strength of this study considering the lack of information in this context. In Angola where data on mortality is based on hospital and government reports, only the number of people dying under medical care is accounted for and death certificates lack accuracy [12]. However it is of crucial importance to know the causes associated to all deaths [52, 53]. Bengo Province, where the study area is located, is reported as an endemic region for several infectious diseases responsible for morbidity and mortality in Angola [14, 31], however there is a very limited capacity of the health structures of registering and measuring the impact of that endemicity on mortality.

The estimated crude mortality for the 3 years encompassing the study period, suggests a decrease in rates, which is in line with the WHO estimates for the same period [11]. However, the estimated crude mortality rate for 2012 that we observed (6.6 per 1000, 95 % CI: 5.9–7.2) may be underestimated: during this period, only one update HDSS round was performed, as opposed to the usual biannual rounds, thus fewer events were reported explaining the observed decrease.

In our study, 18.3 % of deaths were IND, 8.7 % for child and 27.5 % for all adult deaths. This figure lies between the results found in other sub-saharan African countries, namely in Ethiopia (reporting VA deaths for the period 2009–2013) [5] where 14.9 % of deaths were IND and a 2012 study from Tanzania reporting 31 % deaths with indeterminate causes [33]. The high proportion of IND causes of death is a common limitation attributed to the VA system, that may result from the relatively low specificity and sensitivity of the VA tool for detecting some causes of death [4, 29], and from other influencing factors such as the interaction between interviewer and respondent, the setting where the VA is performed, recall bias and the subjectivity inherent to the physician review [1]. Further investigation of what determines indeterminate causes is warranted.

One other problem felt in the Dande HDSS, is the scarcity of physicians available to be trained to read VA questionnaires. In the future, the validity of the causes of death derived from VA reviewed by physicians should be tested with the use of a probabilistic approach, such as the InterVA method [1].

## Conclusions

The findings of this study confirm international estimates on the main causes of death and provide a more detailed picture of mortality in this region, informing health policies and focused interventions. The VA system proves to be a useful tool in this context, where almost half of deaths occur outside a health facility and mortality data is scarce and often inaccurate.

Preventive measures should be given priority, since infectious diseases remain the major cause of death, despite the country’s development in the past decade. Simultaneously, efforts tackling NCD and INJ cannot be neglected and should initiate immediately in order to prevent escalation of its burden.

## Abbreviations

CD, communicable diseases; CISA, Health Research Centre of Angola; CoD, causes of deaths; GBD, Global Burden of Disease; HDSS, health and demographic surveillance system; HIV, imunodeficiency virus; ICD-10, 10th revision of the International Classification of Diseases; IND, indeterminate; INEA, National Institute of Statistics of Angola; INJ, injuries; IRB, institutional review board; NCD, Non-communicable diseases; PCA, principal component analysis; SEP, socioeconomic position; TB, tuberculosis; VA, verbal autopsies; WHO, World Health Organization

## Additional file

Additional file 1: Table S1. Proportion of deaths with and without a Verbal autopsy reviewed. Table S2 Distribution death attributable do communicable diseases according to demographic and socioeconomic characteristics. (DOCX 17 kb)
