# Supplementary material for: Main causes of death in Dande, Angola: results from Verbal Autopsies of deaths occurring during 2009–2012
Source: BMC Public Health. 2016 Aug 4;16:719. doi: 10.1186/s12889-016-3365-6 (PMC4973533; doi:10.1186/s12889-016-3365-6)
Supplement: Additional file 1: Table S1. — Proportion of deaths with and without a Verbal autopsy reviewed. Table S2 Distribution death attributable do communicable diseases according to demographic and socioeconomic characteristics. (DOCX 17 kb) [file 12889_2016_3365_MOESM1_ESM.docx]

**Additional file 1**

Table S1. Verbal autopsies reviewed with and without an assigned cause.

|  |  | **Verbal Autopsy** | |  |
| --- | --- | --- | --- | --- |
|  |  | **No**  **n (%)** | **Yes**  **n (%)** | **p** |
| Sex | Female | 308 (44.8) | 368 (46.1) | 0.658 |
|  | Male | 379 (55.2) | 430 (53.9) |  |
| Age | <28 days | 18 (2.6) | 27 (3.4) | <0.001 |
|  | 28 days – 11 months | 44 (6.4) | 124 (15.5) |  |
|  | 1-4 years | 116 (16.9) | 182 (22.8) |  |
|  | 5-14 years | 55 (8.0) | 58 (7.3) |  |
|  | 15-49 years | 223 (32.5) | 200 (25.1) |  |
|  | 50+ years | 231 (33.6) | 207 (25.9) |  |

Table S2 - Distribution death attributable do communicable diseases according to demographic and socioeconomic characteristics

|  |  | **Nutritional conditions** | **Maternal causes** | **Perinatal causes** | **Other Communicable Diseases** |  |
| --- | --- | --- | --- | --- | --- | --- |
|  |  | **n (%)** | **n (%)** | **n (%)** | **n (%)** | **p** |
| **Sex** | **Female** | 35 (54.7) | 9 (100) | 9 (50.0) | 225 (47.0) | 0.012 |
|  | **Male** | 29 (45.3) | - | 9 (50.0) | 254 (53.0) |  |
| **Age (years)** | **< 5** | 61 (95.3) | - | 18 (100) | 320 (66.8) | <0.001 |
|  | **5-14** | 3 (4.7) | - | - | 41 (8.6) |  |
|  | **15-49** | - | 9 (100) | - | 62 (12.9) |  |
|  | **50+** | - | - | - | 56 (11.7) |  |
| **Education**† **(years)** | **None** | 24 (40.7) | 3 (33.3) | 6 (33.3) | 169 (38.1) | 0.987 |
|  | **1 to 4** | 22 (37.3) | 3 (33.3) | 8 (44.4) | 169 (38.1) |  |
|  | **5 or more** | 13 (22.0) | 3 (33.3) | 4 (22.2) | 106 (23.9) |  |
| **SEP Index** | **Lowest** | 12 (18.8) | 2 (22.2) | 1 (5.6) | 106 (22.1) | 0.265 |
|  | **Low** | 19 (29.7) | 1 (11.1) | 2 (11.1) | 121 (25.3) |  |
|  | **Medium** | 13 (20.3) | 2 (22.2) | 6 (33.3) | 87 (18.2) |  |
|  | **High** | 15 (23.4) | 2 (22.2) | 3 (16.7) | 87 (18.2) |  |
|  | **Highest** | 5 (7.8) | 2 (22.2) | 6 (33.3) | 78 (16.3) |  |
| **Residence** | **Rural** | 8 (12.5) | 2 (22.2) | 4 (22.2) | 74 (15.4) | 0.711 |
|  | **Urban** | 56 (87.5) | 7 (77.8) | 14 (77.8) | 405 (84.6) |  |
| **Place of death** | **Health facility** | 35 (54.7) | 4 (44.4) | 8 (44.4) | 291 (60.8) | 0.327 |
|  | **Other/unknown** | 29 (45.3) | 5 (55.6) | 10 (55.6) | 188 (39.2) |  |

p: p-value – chi square test;

†For participants under 15 years of age, the mother’s educational level was considered.

SEP index: socioeconomic position index.
